# Optimization of Evaporation and Condensation Architectures for Solar-Driven Interfacial Evaporation Desalination

**DOI:** 10.3390/membranes12090899

**Published:** 2022-09-18

**Authors:** Cheng Pan, Yawei Yang, Mingze Xie, Qingyuan Deng, Xiang Cheng, Xianlei Wang, Shihan Zhao, Yumeng Wei, Wenxiu Que

**Affiliations:** Electronic Materials Research Laboratory, Key Laboratory of the Ministry of Education, International Center for Dielectric Research, and Shaanxi Engineering Research Center of Advanced Energy Materials and Devices, School of Electronic Science and Engineering, Xi’an Jiaotong University, Xi’an 710049, China

**Keywords:** solar-driven interfacial evaporation, seawater desalination, condensation, collection efficiency

## Abstract

Solar-driven interfacial evaporation is an ideal technology for seawater desalination, and the corresponding system is mainly composed of a solar evaporator and a condensing collector. The traditional scheme focuses on the evaporation efficiency of the evaporator. Still, it ignores the influence of condensing collection scheme on the overall efficiency, which is one of the obstacles to the practical use of solar seawater desalination. Here, we reported a new solar-driven interfacial evaporation seawater desalination system by studying the influence of the condensation architecture, i.e., vapor flow by a fan and an air pump, sidewall material, transparent cover shape and material, evaporation level, and transparent cover heating, on the apparent collection efficiency of the system. The apparent collection efficiency was up to over 90% after optimization. This study is expected to promote the practical application of solar evaporation desalination technology.

## 1. Introduction

The freshwater resource shortage is becoming more severe due to global warming and environmental pollution. It has been predicted that by 2025, about 60% of the population in 48 countries and regions worldwide will face a shortage of freshwater resources, and freshwater will become a contested strategic resource by all countries after oil [1]. Unlike traditional evaporation systems based on overall heating, solar-driven interfacial evaporation systems consist of an evaporator and a condensing collector. Firstly, a small amount of water is isolated from the water below, thus limiting the solar–thermal conversion to the air-isolated water interface. Additionally, only the water at the interface is heated and evaporated, significantly reducing the heat loss, and water vapor can be easily obtained. This method easily achieves a photothermal–vapor conversion efficiency of more than 80% [2], allowing the vapor to be condensed and collected as freshwater. However, for the standard photothermal/evaporation interfacial integrated evaporator, it is often necessary to place a transparent cover above it for light transmission and vapor condensation collection.

The existing literature is mainly focused on the research of interfacial evaporators and is very seldom related to the design of condensing collectors. Moreover, the structure of such a common interfacial evaporator has three congenital shortcomings. Firstly, the vapor mist and water droplets condensed on the transparent top cover usually lead to severe light loss (up to 35%); secondly, due to the requirement of optical transparency, the top cover is generally made of polymer (such as acrylic) or glass, while these materials have typically 5–20% of the optical loss due to the limitation of their transmittance, and their low thermal conductivities (K) (K < 5 W/m K) [3] are not conducive to the process of liquefied heat release required by vapor condensation; thirdly, direct sunlight and vapor liquefaction heat release will increase the temperature of the top cover and further affect the subsequent condensation effect [4]. Thus, the device integrated solar-collected water conversion efficiency is usually only about 35% [5,6].

Based on the above problems, and considering that the condensing collector is the key to affecting the comprehensive solar energy conversion efficiency of solar-driven interfacial evaporation technology, an ideal solar-driven interfacial evaporation seawater desalination system was put forward and achieved by optimizing the condensation architecture and material. The apparent collection efficiency is defined as water collected in a closed system divided by the evaporation in the open system. The maximum apparent collection efficiency of 98.97% of the as-fabricated system in the laboratory was obtained by optimizing airflow disturbance, transparent cover architecture, evaporation level, and light transmission. This work aims to optimize the evaporation and condensation structures, obtain a better collection efficiency, and obtain a high-efficiency desalination system. These results indicate that this work lays a foundation for applying solar-driven interfacial evaporation seawater desalination.

## 2. Experimental Section

### 2.1. Preparation of the Photothermal Membrane

**Figure 1:**
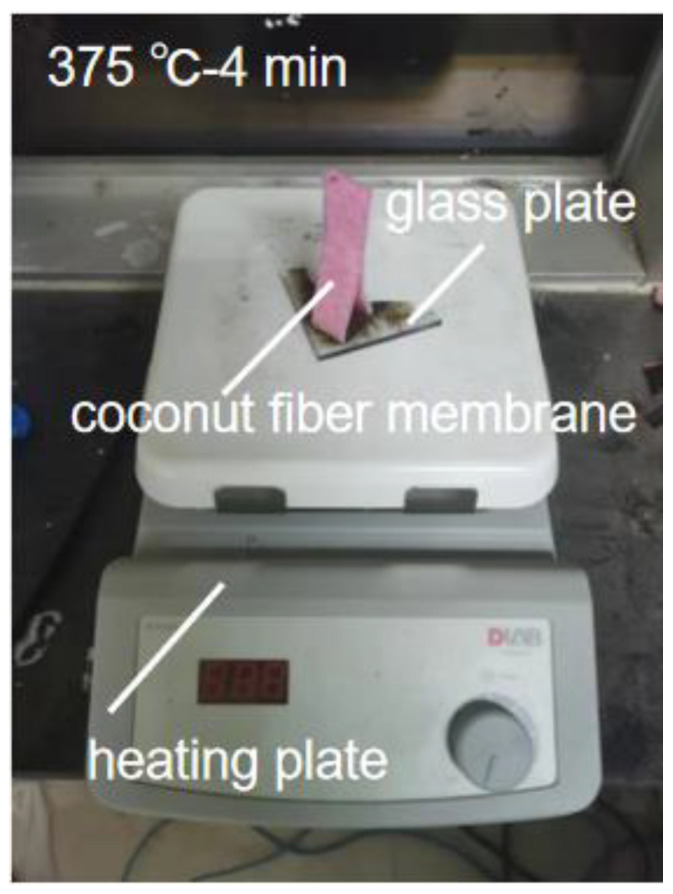
Carbonization of the coconut fiber membrane.

A heating plate was applied for carbonizing the coconut fiber membrane. First, cut the coconut fiber membrane (commercially available from Mengju Han company, China) to a proper size, and preheat the heating plate (HP550-S, Beijing, China), which is covered by a piece of glass, to the target temperature (325–400 °C) before carbonization. Second, place the coconut fiber membrane on the preheated glass for 3–5 min (Figure 1). Carbonize one surface of the coconut fiber membrane until it completely turns black. It should be noted that excessive carbonization is not allowed because, as a photothermal layer, the coconut fiber membrane needs to maintain a certain mechanical strength and stability (Supplementary Materials, Figure S1). For evaporators, the price of coconut fiber membrane is ~1.0 USD/m^2^, and simple carbonization operation does not significantly increase the cost.

### 2.2. Solar Evaporation Performance of the Photothermal Membrane

**Figure 2:**
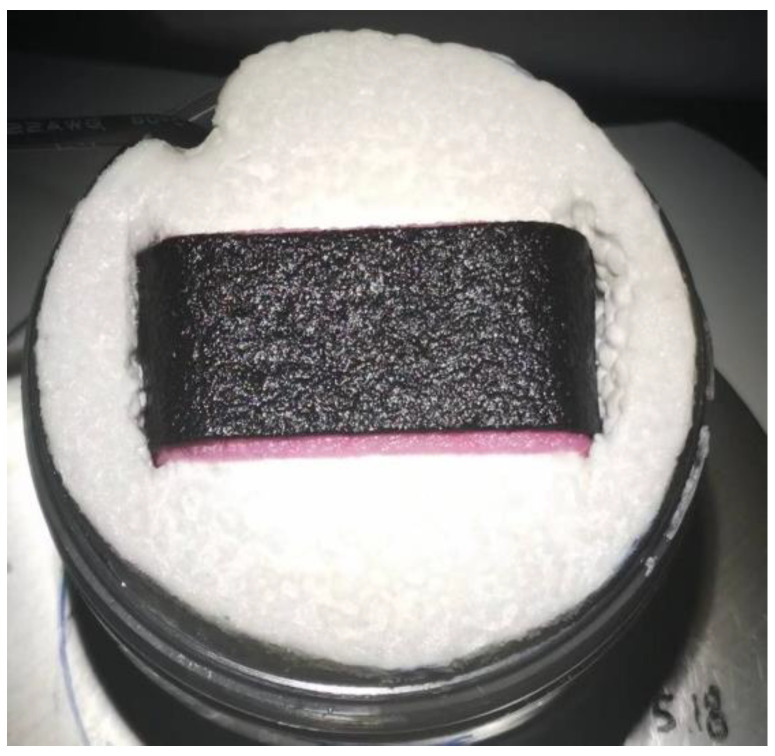
Carbonized coconut fiber membrane-based photothermal layer as a solar evaporator.

Fix the photothermal membrane on polystyrene foam, and the uncarbonized part is immersed in water, working as a water supply channel. That is the solar evaporator (Figure 2), which was put on top of a container. In the solar evaporation test, the simulation sunlight was generated by a xenon lamp (PLS-SXE300, Beijing PefectLight, Beijing, China) with an AM 1.5 G filter. The light intensity was calibrated to 3 suns (3 kW/m^2^) by a light intensity meter (CEL-NP2000-2). Natural sunlight has an intensity of 0.5~2 kW/m^2^ in different regions of the earth. An intensity of 3 suns was applied considering the potential focus light condition. An electronic scale monitored the mass loss of the simulation seawater in real time (NV422ZH, OHAUS, Parsippany, NJ, USA, accuracy in 0.01 g). An IR camera (CAT-S60) was used to identify the surface temperature of the evaporator.

### 2.3. Optimization of Vapor Flow and Operational System Design

First, the photothermal layer was set to a size of 3.2 × 3.3 cm^2^. Then, the container with the solar evaporator was put in the bottom of a sealed plastic/aluminum cylinder with a flat top window, which is adopted as the vapor condenser and freshwater collector (Supplementary Materials, Figure S2). For the blow collection scheme (vapor condensation by blowing to the side wall), a fan is fixed at a position a little higher than the photothermal membrane, with the front side facing the vapor and the back side having a gap to the side wall. For the pump collection scheme (vapor collection by pumping out), an air pump was applied by connecting to a pipe inserted into the closed system. For the blow + pump collection scheme, both a fan and an air pump were simultaneously applied. Aluminum has good thermal conductivity, which is expected to enhance condensation performance. For the collector, ready-made plastic or metal cylinders are used. The price of plastic and aluminum cans is ~0.4 USD/L and 1.0 USD/L, respectively.

### 2.4. Optimization of the Transparent Cover Architecture and Evaporation Level

A bigger solar desalination system was developed for practical use. Four pieces of 6.5 × 6.5 cm^2^ photothermal membranes were used as a solar evaporator. In sequence, the evaporator height was adjusted to low, moderate, and high levels, nearer to the top cover. A commercially available aluminum pot was applied as a freshwater collector. The light intensity was calibrated to 1 sun (1 kW/m^2^). We put the evaporator on a polystyrene foam container, which was set in the center of the aluminum pot. The droplets on the transparent cover scatter light heavily. Hence, optimizing the transparent cover architecture is necessary to avoid serious optical loss. Samples of typical commercially available transparent covers with different shapes and materials, including spherical plastic cover, flat glass cover, small radian glass cover, and moderate radian glass cover, were employed as the top cover. We sealed the top cover and the aluminum pot with a silicone loop to prevent vapor from escaping. No fan and air pump were applied in these cases.

### 2.5. Optimization of the Light Transmission

The location of the droplet condensation can be controlled through an energy gradient. Hence, top cover heating was proposed to adjust the energy gradient for driving the vapor to the side wall for condensation. Active and passive heating wires were employed to prevent the water droplets on the top cover. The heating wires were pasted on the outer surface of the transparent cover in a circle, which provides a uniform heat distribution on the cover. The active heating wire is a resistance wire heated by electricity, while the passive one is a light-absorbing wire heated by solar irradiation.

### 2.6. Characterizations

**Figure 3:**
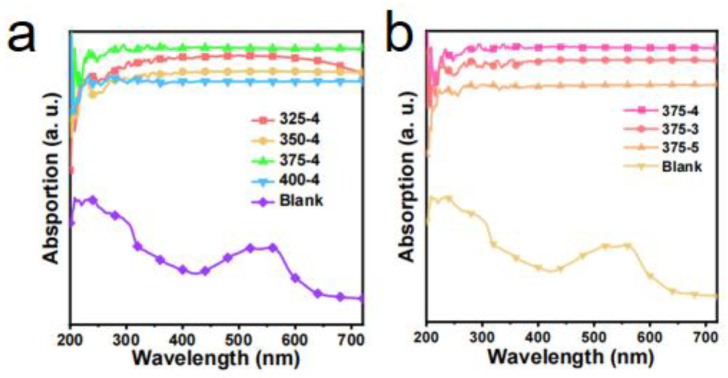
UV-Vis absorption spectra of the carbonized coconut fiber membranes: (**a**) different carbonized temperatures for 4 min and (**b**) different carbonized times at 375 °C.

**Figure 4:**
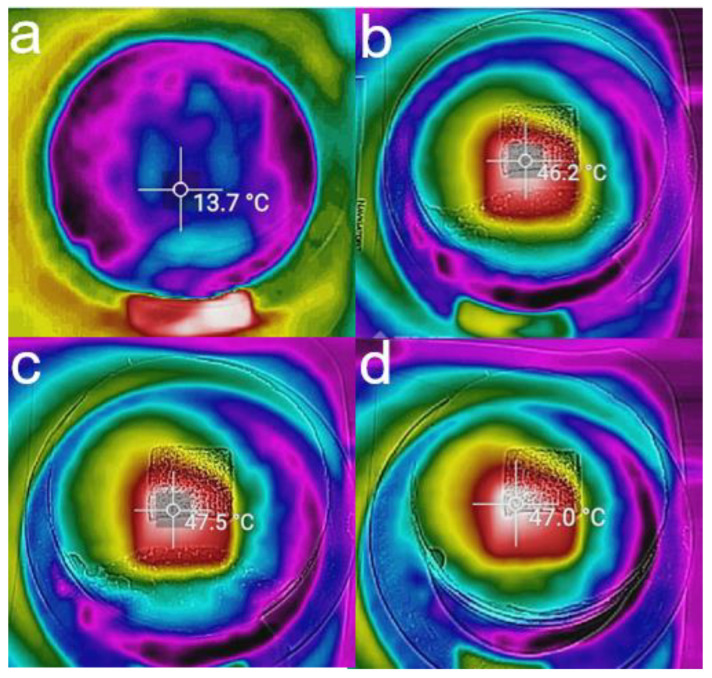
IR image of the carbonized coconut fiber membrane (375 °C, 4 min) under 3 suns: (**a**) initial, (**b**) 5 min, (**c**) 1 h, and (**d**) 2 h.

The morphology of the samples was observed by a scanning electron microscope (SEM, Quatan 250FEG, FEI, Hillsboro, OH, USA). The light absorption of the samples was measured by a UV-Vis spectrometer (Jasco, V570, Tokyo, Japan). An IR camera was used to identify the surface temperature of the evaporator (CAT-S60). The carbonized coconut fiber membrane’s UV-Vis absorption spectra (Figure 3) and IR images (Figure 4) show excellent photothermal conversion ability.

## 3. Results and Discussion

### 3.1. Evaporation Performance of the Solar Evaporator

**Figure 5:**
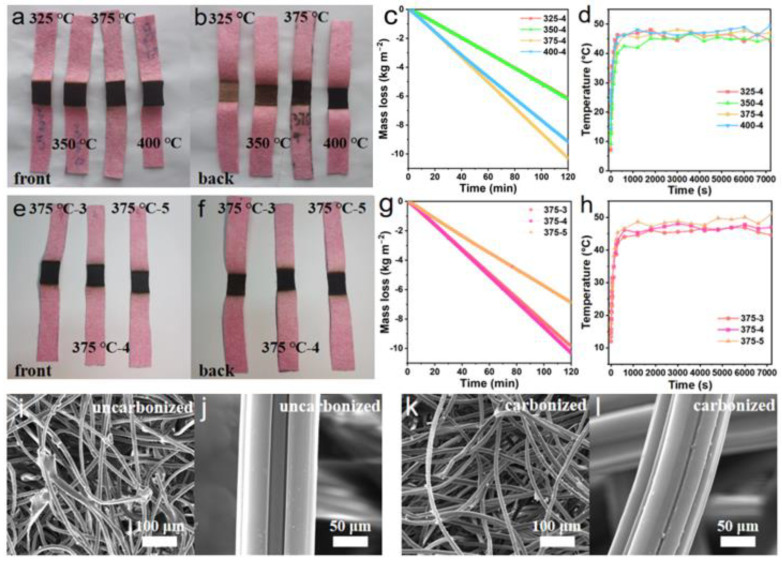
Effect of carbonization time and temperature on solar evaporation performance: coconut fiber membrane carbonized at different temperatures: (**a**) front view and (**b**) back view. (**c**) Solar evaporation performance and (**d**) surface temperature of coconut fiber membrane treated at different carbonization temperatures. Coconut fiber membrane carbonized at different times: (**e**) front view and (**f**) back view. (**g**) Solar evaporation performance and (**h**) surface temperature of coconut fiber membrane treated with different carbonization times. SEM images of (**i**,**j**) uncarbonized and (**k**,**l**) carbonized coconut fiber membranes.

Solar water desalination consists of evaporation [7,8,9,10,11,12,13,14], salt resistance [15,16], heat insulation [9,10,17,18,19], water transport channel [20,21], and vapor collection [22,23,24,25]. The performance of the evaporator is the key to the comprehensive performance of solar water desalination. At present, there have been many studies on the evaporator. The structure of the evaporator has experienced development from one to three dimensions. The material of the evaporator also includes a carbon-based material [26], semiconductor material [27,28,29], or metal nanoparticles with plasma absorption [30,31,32]. The optimization of evaporator evaporation efficiency is the basis of efficient condensation collection. Therefore, the optimization experiment of the evaporation layer is carried out. The coconut fiber membrane is mainly composed of cellulose, lignin, and pentosan. Through carbonization, it turns to amorphous carbon fiber (Supplementary Materials, Figure S3), which is conducive to the absorption of the sunlight spectrum. Therefore, a hydrophilic coconut fiber membrane is used as the water transport channel, and it can be carbonized to obtain the light-absorbing material as the photothermal layer of the evaporator. It can control the temperature variables, creating 325 °C, 350 °C, 375 °C, and 400 °C carbonized samples for four minutes, through the experimental results show that in a certain temperature range, the carbonization temperature is higher, the photothermal effect of the evaporation layer is better (Figure 5a,b).

The process of evaporation is tested for two hours under 3 suns. The mean surface temperatures of 325 °C, 350 °C, 375 °C, and 400 °C were increased to 46.03 °C, 44.63 °C, 46.78 °C, and 47.39 °C, and their evaporations were 3.09, 3.10, 5.15, and 4.59 kg m^−2^ h^−1^ in the open environment, respectively (Figure 5c,d). However, if the temperature is too high, it will also make the carbonization photothermal layer fragile. Under the carbonization temperature of 375 °C, the photothermal layer has better evaporation performance. Therefore, 375 °C is selected as the optimal carbonization temperature. Based on the above results, it can also control the time variable. Carbonation time is another critical parameter affecting the photothermal layer performance. If the heating carbonization time is too short, it will result in insufficient heating of the coconut fiber membrane and will not produce a light absorption effect; if the heating carbonization time is too long, the thickness of the coconut fiber membrane will be reduced, so that the mechanical properties will be worse. The performance of the evaporator will also be affected, and the working life of the photothermal layer will be reduced. Under the carbonization temperature of 375 °C, when the time interval is set to 3, 4, and 5 min, respectively, the experimental results show that if the coconut fiber membrane is carbonized longer, the carbonization effect would also be better (Figure 5e). It can be seen that the carbonization region becomes thinner with the increase in carbonization time (Figure 5f). The properties of coconut fiber membrane with carbonization times of 3, 4, and 5 min are tested, and the evaporation performance is tested for two hours under 3 suns. Under 3 suns, the surface temperature of carbonized coconut fiber membrane increases to 45.71 °C, 46.78 °C, and 48.67 °C, and their evaporations are 4.90, 5.15, and 3.44 kg m^−2^ h^−1^ in the open environment, respectively (Figure 5g,h). When the carbonization time is 4 min, the photothermal layer has better evaporation performance. However, the photothermal layer’s evaporation performance after five minutes of carbonization decreased by nearly 40%. The reason is that if the carbonization time is longer, the carbonization effect is better, which results in better light absorption performance. Still, at the same time, the fibrous structure is not conducive to the transportation of water, resulting in the decline of evaporation performance. It can be concluded that a higher carbonization temperature is beneficial to the light and heat absorption properties of the material, but it will also affect the water absorption properties. Therefore, we need to measure the relationship between the variable and get a better carbonization scheme. An SEM test is conducted on the coconut fiber membrane before and after carbonization at 375 °C-4 min (Figure 5i–l). The test results show no significant difference between the coconut fiber membrane after carbonization and the original coconut fiber membrane (Figure 5k). So the coconut fiber membrane’s porous and porous fiber structure is not changed by heating the coconut fiber membrane. That is why heating coconut fiber membrane can be used as a photothermal conversion layer and water transport channel.

### 3.2. Improve Freshwater Collection by Vapor Flow and Operational System Design

**Figure 6:**
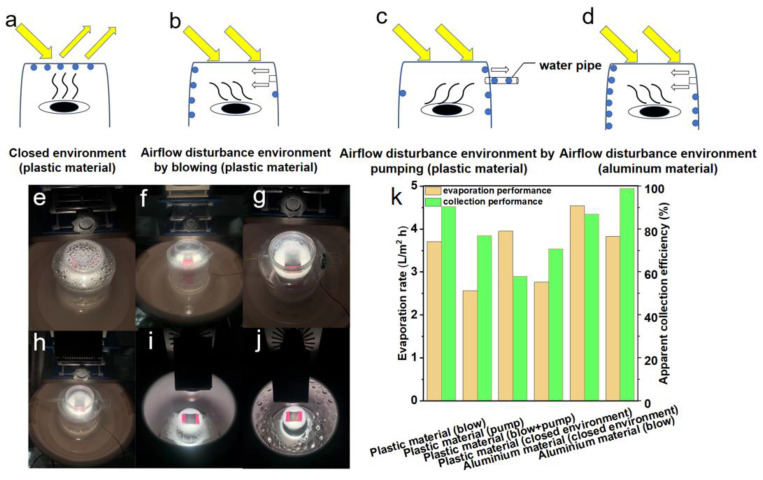
Scheme and solar vapor collection performance of different collection schemes. (**a**) Normally closed environment with serious optical loss. (**b**) Airflow disturbance by blowing with a fan. (**c**) Airflow disturbance by pumping with an air pump. (**d**) Enhancing condensation by high thermal conductivity aluminum side wall. Photos of corresponding collection schemes: (**e**) plastic closed environment, (**f**) blow, (**g**) pump, (**h**) blow + pump, (**i**) aluminum side wall, and (**j**) aluminum side wall with blow. (**k**) Solar evaporation rate and apparent collection efficiency of different collection schemes.

When the carbonization temperature is 325 °C, the coconut fiber membrane has a better balance of mechanical strength and evaporation performance than when the carbonization temperature is 375 °C (Supplementary Materials, Figure S4). Therefore, a coconut fiber membrane with a carbonization temperature of 325 °C is used as the photothermal layer in the condensation optimization experiments. Firstly, a circular tank 9.5 cm in height is used as a water storage device. The carbonized coconut fiber membrane is used as a photothermal layer material. In the optimization architecture of the solar seawater desalination system, the shape of the photothermal layer is square. It has a size of 3.2 × 3.3 cm^2^, a polystyrene foam board with the effect of heat insulation is used as the fixed plate for the photothermal layer, and the fixed plate works as a water transport layer. The fixed plate needs to be tightly fitted to the circular tank to prevent the vapor from flowing back into the water below. A sealed plastic shell was adopted as the vapor collecting unit, and a square window (2.5 × 2.5 cm^2^) was opened on the side wall of the shell to fix the fan in the opening. On the opposite side of the square window, a circular opening with a diameter of 1 mm is needed to fix the catheter of the micro pump through the opening. Based on the above design and optimization, the contrast experiments, which include the airtight environment, flow disturbance, micro pump suction, and airflow disturbance + micro pump suction, are carried out (Figure 6a–d). Results indicate that in a closed environment, many condensed droplets are attached to the surface of the incident light cover, while a large amount of light is refracted by the droplets. As a result, the photothermal layer does not have enough heat to generate vapor, thus affecting the continuous evaporation process (Figure 6e).

The experimental results after 24 h show that the evaporation volume is 70 mL, the collection volume is only 49.5 mL, and the apparent collection efficiency is 70.71%. To overcome the above light absorption problem, a 0.75 W electric fan was used to disturb the airflow and form a disturbance layer above the evaporation layer, which drives water vapor to diffuse to the side wall (Figure 6f). Thus, it can prevent vapor condensation in the transparent layer, and sunlight can easily reach the photothermal layer. After the 24 h test, the evaporation volume increases to 94 mL, and the collection volume also increases to 85 mL. The apparent collection efficiency achieves 90.43% with an electrical fan. It indicates that the airflow disturbance drives the vapor direction. Additionally, there are almost no condensation droplets on the surface of the incident light cover, which dramatically improves the efficiency and stability of the vapor generation and collection process. In addition, the micropump is used to extract the vapor. Results show that after a 24 h test, the evaporation volume is 65 mL, the collection volume is only 50 mL, and the apparent collection efficiency is 76.92% (Figure 6g). It can be seen that the evaporation volume of the micro pumping scheme is lower than that of the closed scheme. Vapor cannot be efficiently collected by the pumping scheme, and is not conducive to evaporation.

Furthermore, combining airflow disturbance with air pumping was also attempted. The results indicate that the evaporation volume and collection volume of the 24 h experiment are 100 mL and 58 mL, respectively, and the apparent collection efficiency is further decreased to 58.00% (Figure 6h). The reason for the evaporation decrease is that the pumping method disturbs the flow direction of vapor, which causes the vapor pressure to rise and causes secondary evaporation problems. The collection amount is also reduced due to the failure of the vapor to condense on the side wall in time. The decrease in collection volume has resulted in higher humidity in the evaporation chamber, as the water in the air has reached saturation, so the apparent collection efficiency has also decreased. Based on the above experimental results, an apparent collection efficiency of 90.43% can be obtained by using only the airflow disturbance scheme.

With its better thermal conductivity, aluminum can be used to carry out comparative experiments. The first test is performed under entirely confined conditions, also at 3 suns. After the 24 h investigation, the evaporation volume is 115 mL, the collection volume is 100 mL, the evaporation rate is 4.54 L/m^2^ h, and the apparent collection efficiency is 86.96% in the closed environment. Compared with the plastic collection device, the collection performance is improved by 16.25%. There are more condensation droplets on the side wall, and the vapor pressure is also reduced due to timely condensation and secondary evaporation being effectively avoided. It should be mentioned here that the glass cover plate is adopted at the top for light transmission in this scheme. Thus, there is no large amount of water droplets attached in the center of the cover plate due to high energy, but a large number of droplets are attached in the surrounding place with a low temperature. Hence, the collection amount is more significant than that of the plastic cover plate (Figure 6i). Following that, the airflow disturbance unit is set on the side wall of the aluminum tank, in which the electric fan is set 2 cm above the photothermal layer (Figure 6j). The 24 h experiment results show that the evaporation volume is 97 mL, the collection volume is 96 mL, the evaporation rate was 3.83 L/m^2^ h, and the apparent collection efficiency is up to 98.97%, which is superior to the majority of previous work (Supplementary Materials, Table S1). The evaporation rate and apparent collection efficiency of different collection schemes are summarized in Figure 6k. It can be affirmed again that using the airflow disturbance scheme is an effective strategy to solve the problem of vapor condensation and prevent the incidence of light in the top cover. It can be concluded that the material with good thermal conductivity is conducive to vapor condensation, reduces the vapor pressure of the collection chamber, and avoids secondary evaporation. However, the evaporator should also be insulated to ensure stable and efficient water vapor generation.

### 3.3. Improved Freshwater Collection by Adjusting Transparent Cover Architecture and Evaporation Level

**Figure 7:**
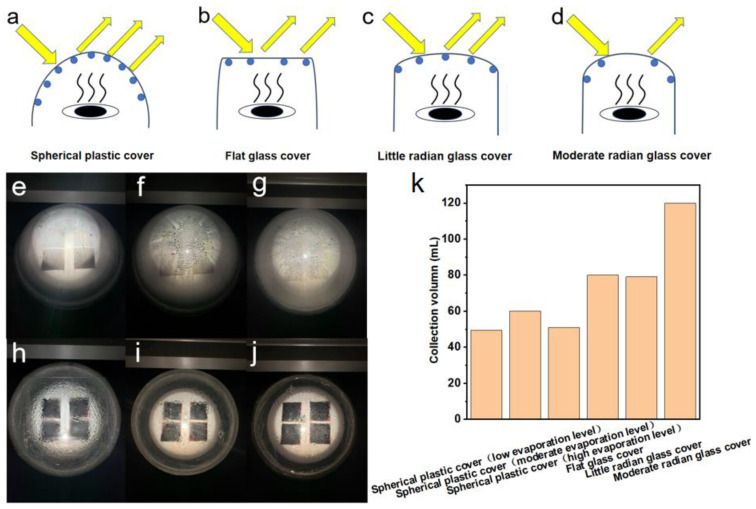
Scheme and solar vapor collection performance of different top covers. (**a**) Spherical plastic cover with serious optical loss. (**b**) Flat glass cover with large droplets. (**c**) Small radian glass covered with large droplets. (**d**) Moderate radian glass cover with mist. Photos of corresponding top covers: spherical plastic cover with (**e**) low, (**f**) moderate, (**g**) high evaporation levels; (**h**) flat glass cover, (**i**) small radian glass cover, and (**j**) moderate radian glass cover with moderate evaporation level. (**k**) Collection performance of different top covers.

Droplet condensation on the cover plate results in a serious light loss of up to 35%, the cover plate material, which is usually made of a polymer such as acrylic or glass, generally has a light loss of 5~20% due to the transmittance limitation of the polymer material, as well, the radian of the cover plate also influences the vapor collection (Figure 7a–d). Hence, it is necessary to optimize the architecture and design of the low-temperature vapor condensation, droplet rolling collection, and latent heat release under the condition of wettability. Thus, the covers with different structures and shapes are used to carry out the experiments. When the nearly spherical plastic cover is used as the collection device, it indicates that the condensation density of the droplets is large and the droplet’s shape is small. The radian is large, which easily reflects the light, but the droplets show the advantage of easy rolling (Figure 7e). Results show that when four small photothermal layers are used to heat the water under 1 sun, the collection volume is 49.5 mL in a day when the spherical plastic is covered with low evaporation levels. It is also noted that when the spherical plastic is covered with moderate evaporation levels, the water amount of the collection increases from 49.5 mL to 60 mL. Because of the large radian of the spherical condensation cover, the droplets are easy to gather at the top, and the plastic material generally affects condensation. Therefore, increasing the height between the evaporation surface layer and the top cover is beneficial to optimize the condensation effect (Figure 7f). However, this effect is not further enhanced for spherical plastic covers with high evaporation levels. Additionally, when the height between the evaporation surface layer and the top cover is further increased, the condensation volume decreases to only 51 mL, which indicates that when the height is too high, the generated vapor cannot condense on the top cover in time (Figure 7g). The increase of the vapor pressure in the collecting chamber will lead to secondary evaporation, which also affects the evaporation rate. Therefore, an optimal setting distance needs to be adjusted according to the actual device.

According to the optimized evaporation levels obtained above, when the flat glass plate is used as the top cover, the collection volume for a day is 80 mL. The attachment of liquid droplets on the flat glass plate is not as dense as that of the spherical cover, which is more conducive to the light passing through the glass to reach the photothermal layer (Figure 7h). When a small radian glass plate is used as a top cover, the amount of the collected water is 79 mL (Figure 7i), which has no significant improvement compared to the flat glass plate. In the subsequent experiment, a moderate radian glass cover is used, and the collection volume is 120 mL in a day (Figure 7j). The collection volume of different covers with different materials and the radian is summarized in Figure 7k. These results indicate that the collective effect of the moderate radian glass cover is the best one, owing to the minimal impact on the light reflection and the suitable rolling property of droplets.

### 3.4. Improve Freshwater Collection by Increasing Light Transmission in a Transparent Cover

**Figure 8:**
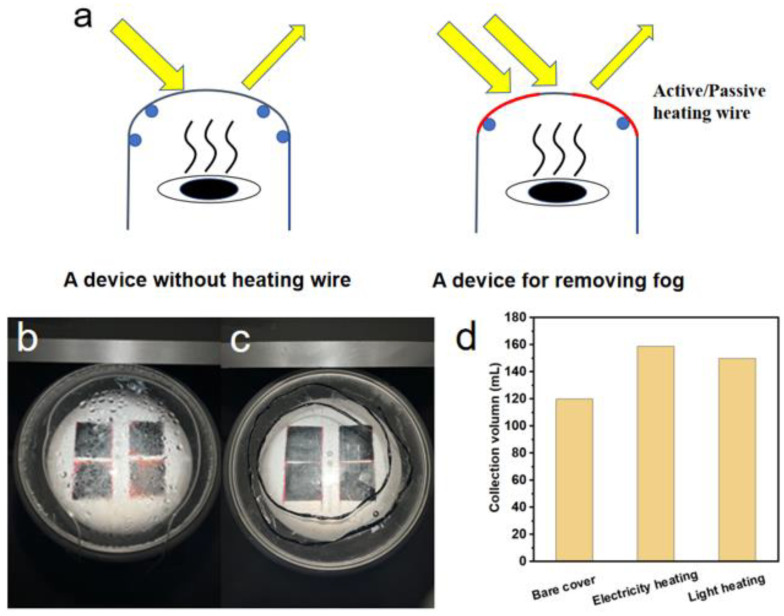
Scheme and solar vapor collection performance of top cover heating: (**a**) bare cover with optical loss and heating wire on the cover with high light transmission. Photos of corresponding heating wires: (**b**) active (electricity) heating wire and (**c**) passive (light) heating wire. (**d**) Collection performance of different cover heating ways.

The condensation of droplets on the top cover is an inevitable problem for the upward evaporation scheme. Droplets not only refract light but are also not conducive to reducing indoor vapor pressure, which seriously affects seawater desalination’s evaporation and collection efficiency. Hence, it is an urgent problem that needs to be solved. Actually, when optimizing the thermal conductivity material, it is also noted that the droplets do not condense in the middle region due to being close to the light source. There are a large number of droplets attached at the edge. This indicates that the location of the droplet condensation can be controlled through the energy gradient (Figure 8a). Thus, active and passive heating wires are used to remove the water droplets, including heating elements and fixed fittings. The heating wire is arranged on the outer surface of the transparent cover in spiral mode with equal spacing on the outer surface and fixed, which is conducive to the uniform distribution of the heating wire energy. The setting of the defogging unit is direct to the sunlight so that the light source can illuminate the surface of the defogging unit as much as possible. Such a setting is expected to effectively increase the heating efficiency. In addition, the defogging unit can also be wrapped with a black light-absorbing material to facilitate the conversion of light and heat energy, which can be used as a passive structure. Using four photothermal layers with an area of 6.5 × 6.5 cm^2^ for evaporation under 1 sun, 159 mL of water can be collected in a day, which is 39 mL more than the natural collection under the same conditions, and the apparent collection efficiency is improved by 1.33 times, showing that the collection effect is significantly enhanced (Figure 8b). In addition, it is also noted that the winding mode of the heating wire also affects the collection effect. When the heating wire is wound at equal spacing, the collection effect is more obvious than that of a single loop, but if the arrangement is too dense, it will affect the incidence of light. When the defogging unit wrapped in black material is used, the collection volume is 150 mL a day, which is not as high as the heating energy scheme of the electric heating wire, but it also has a good effect: the apparent collection efficiency is increased by 1.25 times (Figure 8c). Compared with the active structure of the heating wire, the passive structure has the advantage of energy saving and environmental protection. In particular, the passive defogging structure can be set outdoors. The collection volume of different heating wire schemes is summarized in Figure 8d.

Based on the above analysis and discussion, the material, shape, height, and some other key structural parts of the collection condensation unit; the condition of the low-temperature vapor condensation process; the performance of the droplet rolling collection, and the latent heat release for the solar-driven interfacial evaporation system have been optimized. Additionally, the optimized water-cooled collector can efficiently liquefy vapor and discharge liquid droplets. On this basis, the condensation collection architecture with the characteristics of fast condensation, highly efficient heat conduction, and fast droplet rolling performance is designed and fabricated. It is found that with the addition of the airflow disturbance unit, the collected distilled water increases from 49.5 mL to 85 mL, and the apparent collection amount increases 1.72 times. A metal shell with good thermal conductivity increases the collected water from 49.5 mL to 100 mL, and the corresponding amount is increased by 2.02 times, while the condensation amount of the top glass cover compared to the plastic cover is increased by 2.00 times, and the rolling droplet effect is also better.

## 4. Conclusions

The condensation architecture is the key limitation of collection efficiency for solar interfacial evaporation seawater desalination. Since the current situation of few reports on this issue, the problem of low solar vapor collection efficiency in the present closed system has been greatly solved by optimizing the condensation architecture. The limitation of water droplets on the top cover, which leads to high optical loss, low evaporation rate, and low vapor condensation, has been significantly broken by optimizing vapor flow and condensation side wall architecture, transparent cover architecture, evaporation level, and active/passive cover heating. Finally, a solar desalination system with an ideal apparent collection efficiency of over 90% has been obtained. The fabrication and cost of this design are economically friendly. We believe this work lays a foundation for the practical application of solar seawater desalination.

## Data Availability

The data supporting the reported results of the current study are available from the corresponding authors upon reasonable request.

## Supplementary Materials

The following supporting information can be downloaded at: https://www.mdpi.com/article/10.3390/membranes12090899/s1, Figure S1: Coconut fiber membrane with excessive carbonization: (a) front view, (b) back view, and (c) side view, Figure S2: The sealed plastic and aluminum cylinder (a) with a flat transparent top cover (b), Figure S3: Raman spectra of (a) coconut fiber membrane, and (b) carbonized coconut fiber membrane, Figure S4: Tensile strength-strain curves of the coconut fiber membrane and carbonized membranes at 325 °C and 375 °C, Table S1: Collection efficiency of this work compared with previously reported solar desalination systems. References [13,33,34,35] are cited in Supplementary Materials.
