# Supplementary material for: Optimization of Evaporation and Condensation Architectures for Solar-Driven Interfacial Evaporation Desalination
Source: Membranes (Basel). 2022 Sep 18;12(9):899. doi: 10.3390/membranes12090899 (PMC9506556; doi:10.3390/membranes12090899)
Supplement: Supplementary file 1 [file membranes-12-00899-s001.zip › membranes-1887755-supplementary.pdf]

membranes

Supplementary Materials

Optimization of Evaporation and Condensation Architectures for Solar-Driven Interfacial Evaporation Desalination

Cheng Pan, Yawei Yang *, Mingze Xie, Qingyuan Deng, Xiang Cheng, Xianlei Wang, Shihan Zhao, Yumeng Wei and Wenxiu Que *

Electronic Materials Research Laboratory, Key Laboratory of the Ministry of Education, International Center for Dielectric Research, and Shaanxi Engineering Research Center of Advanced Energy Materials and Devices, School of Electronic Science and Engineering, Xi'an Jiaotong University, Xi'an 710049, China

* Correspondence: ywyang@xjtu.edu.cn (Y.Y.); wxque@xjtu.edu.cn (W.Q.); Tel./Fax: +86-29-83-395-679 (W.Q.)

Citation: Pan, C.; Yang, Y.; Xie, M.; Deng, Q.; Cheng, X.; Wang, X.; Zhao, S.; Wei, Y.; Que, W. Optimization of Evaporation and Condensation Architectures for Solar-Driven Interfacial Evaporation Desalination. *Membranes* **2022**, *12*, 899. https://doi.org/10.3390/membranes12090899

Academic Editor: Elena Kalinina

Received: 12 August 2022
Accepted: 14 September 2022
Published: 18 September 2022

Publisher's Note: MDPI stays neutral with regard to jurisdictional claims in published maps and institutional affiliations.

Copyright: © 2022 by the authors. Licensee MDPI, Basel, Switzerland. This article is an open access article distributed under the terms and conditions of the Creative Commons Attribution (CC BY) license (https://creativecommons.org/licenses/by/4.0/).

Excessive carbonization of coconut fiber membrane will not only weaken the mechanical strength of the surface of the photothermal layer, but also significantly reduce the thickness of the back side (uncarbonized part). The decreased evaporation rate and efficiency in excessively carbonized evaporator can be attributed to the reduced water supply of back side.

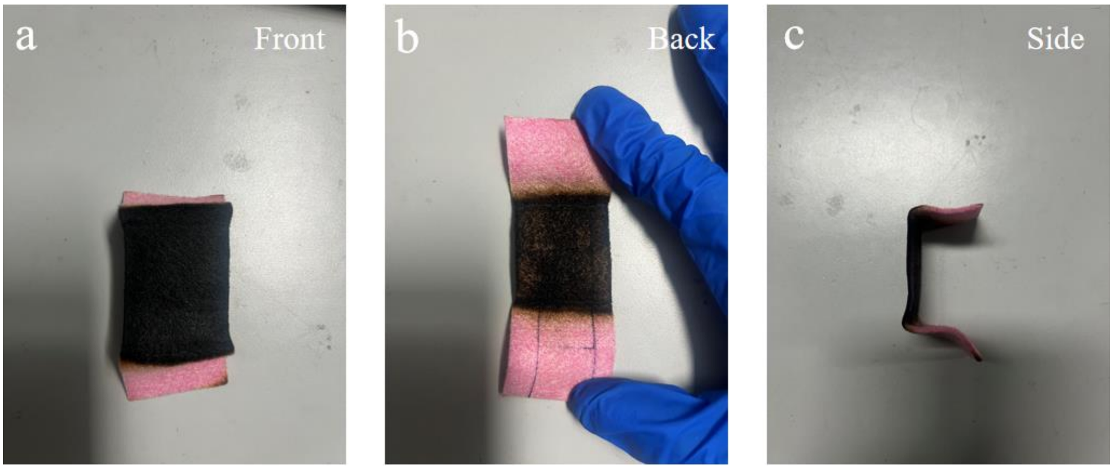

Figure S1. Coconut fiber membrane with excessive carbonization: (**a**) front view, (**b**) back view, and (**c**) side view.

The sealed plastic/aluminum cylinder with a flat top cover is adopted as the vapor condenser and freshwater collector. Transparent top cover is conducive to light transmission. For the blow collection scheme (vapor condensation by blowing to side wall), a fan is fix at a position a little higher than the photothermal membrane, with the front side facing the vapor and the back side having a gap to the side wall. For the pump collection scheme (vapor collection by pumping out), an air pump is applied by connecting to a pipe inserted into the closed system. For the blow + pump collection scheme, both of a fan and an air pump are simultaneously applied. Aluminum has good thermal conductivity, which is expected to enhance the condensation performance.

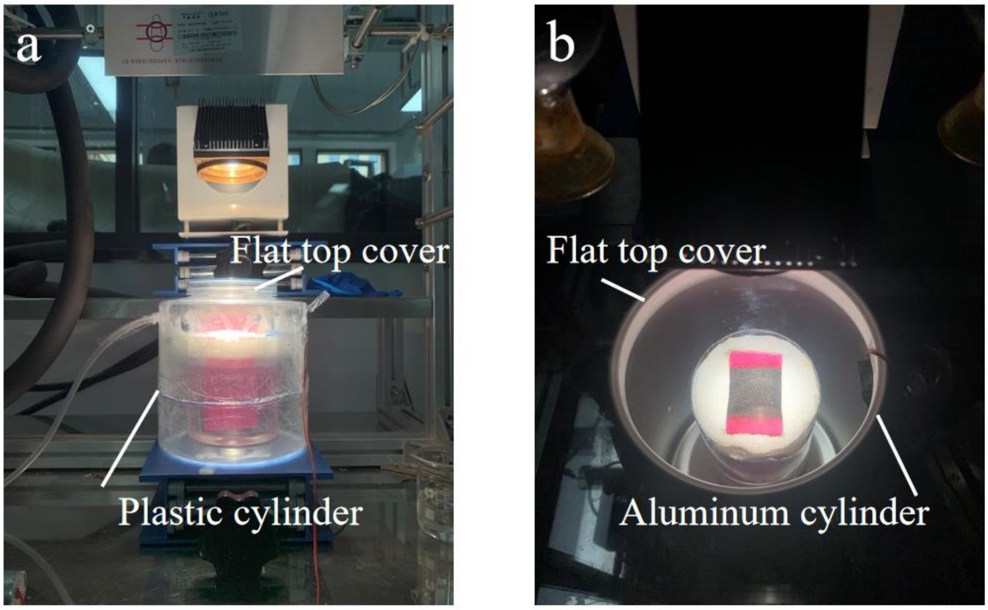

Figure S2. The sealed (**a**) plastic and (**b**) aluminum cylinders with a flat transparent top cover.

The carbonized coconut fiber membrane shows a weak D peak and an obvious G peak of carbon in Raman spectrum. The board Raman peaks indicate the transformation of coconut fiber to amorphous carbon during carbonization.

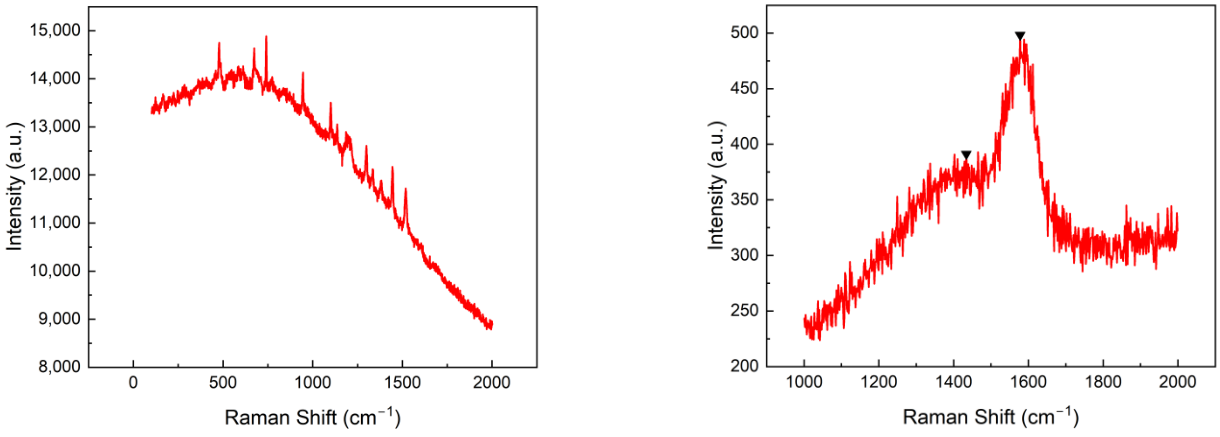

(**a**) (**b**)

Figure S3. Raman spectra of (**a**) coconut fiber membrane, and (**b**) carbonized coconut fiber membrane.

The tensile mechanical strengths of the coconut fiber membrane and carbonized ones were identified and compared. The coconut fiber membrane shows a relatively high elongation over 25% tensile strain. However, the carbonized ones become brittle, and breaks at ~2.5 and ~6.0% tensile strain for 375 °C and 325 °C carbonization, respectively. This result suggests that the 375 °C carbonized membrane is too fragile to use, and the 325 °C carbonized membrane still has enough mechanical strength for solar evaporation.

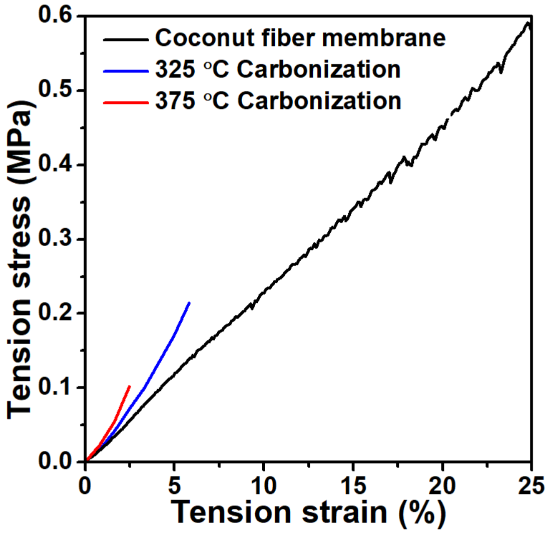

Figure S4. Tensile strength-strain curves of the coconut fiber membrane and carbonized membranes at 325 °C and 375 °C.

Table S1. Collection efficiency of this work compared with previously reported solar desalination systems.

Solar desalination system	Evaporation performance	Collection efficiency	Ref.
Cu_2SnSe_3 nanoparticles on filer membrane/ PMMA box	1.657 kg m^{-2} h^{-1}	45.2% (4h)<br>53.7% (8h)	[1]
TiNOx coated Al plate/ Honeycombed structures of highly thermal-conductive materials with hydrophobic nanostructures	1.063 kg m^{-2} h^{-1}	>70%	[2]
Graphene array coated on commercial nickel foams	73.4%	82.3%	[3]
Commercial spectrally selective solar absorber (B-SX/T-L/Z-Z-1.88,Linuo-Paradigma)/ Graduated cylinder	5.78 L m^{-2} h^{-1} (Ten stages)<br>81% (Single stage)<br>385% (Ten stages)	75%	[4]
This work: coconut fiber membrane/ Optimized collection device	5.15 kg m^{-2} h^{-1}	>90%	/

References

1. Yang, Y.; Zhao, H.; Yin, Z.; Zhao, J.; Yin, X.; Li, N.; Li, D.; Lei, B.; Du, Y.; Que, W. A general salt-resistant hydrophilic/hydrophobic nanoporous double layer design for efficient and stable solar water evaporation distillation. *Mater. Horiz.* **2018**, *5*, 1143–1150.
2. Wang, F.; Xu, N.; Zhao, W.; Zhou, L.; Zhu, P.; Wang, X.; Zhu, B.; Zhu, J. A high-performing single-stage invert-structured solar water purifier through enhanced absorption and condensation. *Joule* **2021**, *5*, 1602–1612.
3. Gong, B.; Yang, H.; Wu, S.; Xiong, G.; Yan, J.; Cen, K.; Bo, Z.; Ostrikov, K. Graphene array-based anti-fouling solar vapour gap membrane distillation with high energy efficiency. *Nano-Micro Lett.* **2019**, *11*, 1–14.
4. Xu, Z.; Zhang, L.; Zhao, L.; Li, B.; Bhatia, B.; Wang, C.; Wilke, K.L.; Song, Y.; Labban, O.; Lienhard, J.H.; Wang, R.; Wang, E. Ultrahigh-efficiency desalination via a thermally-localized multistage solar still. *Energy Environ. Sci.* **2020**, *13*, 830–839.
